# Anxiety in autistic individuals who speak few or no words: A qualitative study of parental experience and anxiety management

**DOI:** 10.1177/1362361320962366

**Published:** 2020-10-01

**Authors:** Joanne Tarver, Effie Pearson, Georgina Edwards, Aryana Shirazi, Liana Potter, Priya Malhi, Jane Waite

**Affiliations:** 1 Aston University, UK; 2 University of Birmingham, UK

**Keywords:** anxiety, autism spectrum disorders, qualitative research

## Abstract

Anxiety is a common co-occurring condition in autism and impacts quality of life of autistic individuals and their families; autistic individuals who speak few or no words represent an under-researched group. This qualitative study aimed to understand more about parental recognition and management of anxiety in autistic individuals who speak few or no words. Semi-structured interviews were conducted with parents/carers of 17 autistic individuals (m_age_ = 14.29) recruited from an existing participant database and social media adverts. Using thematic analysis, 15 themes were placed under three a-priori grand themes: parental recognition of anxiety; parental management of anxiety; and anxiety impact on the autistic individual and their family. Due to reduced verbal language use and overlap with other behaviours, parents described difficulties recognising anxiety in their child. However, they also described use of a number of management strategies, including some which overlap with components of evidence-based interventions for emotional and behavioural problems in autistic individuals (e.g. exposure/sensory calming). Despite this, parents reported that anxiety continues to have significant impact on quality of life. The findings of this study can help to inform the development of targeted intervention and assessment measures for anxiety in autistic individuals who speak few or no words.

**Lay abstract:**

Anxiety is a common condition in autistic individuals, including those who also have an intellectual disability. Despite this, autistic individuals who have severe to profound intellectual disability, or use few or no words, are often excluded from autism research. There are also very few assessment tools and interventions with known effectiveness for autistic individuals with intellectual disability. In this study, we aimed to learn more about parent/carers experiences of recognising and managing anxiety in autistic individuals who use few or no words. We conducted semi-structured interviews with parents and carers to address three research questions: (1) what techniques and management strategies do parents describe for anxiety-related behaviour in their child; (2) how do communication difficulties impact parental understanding and management of anxiety provoking situations and behaviours; (3) what is the impact of anxiety-related behaviours on the quality of life of autistic individuals and their families? During the interviews, parents described difficulties recognising anxiety in their child, mostly due to reduced verbal language use and anxiety behaviours overlapping with other behaviours (e.g. autism characteristics). However, parents also described use of a number of management strategies, including some which overlap with components of evidence-based interventions for emotional and behavioural problems in autistic individuals (e.g. exposure/sensory calming). Despite this, parents reported that anxiety continues to have significant impact on quality of life. We will use the findings of this study to inform future research to develop assessment tools and interventions for anxiety in autistic individuals who use few or no words.

Autism spectrum disorder (ASD) is a neurodevelopmental condition characterised by differences in social communication skills, restrictive and repetitive behaviour and sensory processing difficulties (American Psychiatric Association, 2013). Mental health difficulties are highly prevalent in autistic individuals, with anxiety disorders among the most common co-occurring conditions; prevalence rates for anxiety disorders in autism have been estimated around 20%–40% (Lai et al., 2019; Simonoff et al., 2008).

While anxiety in autistic individuals can present in traditional ways, mapping onto standard definitions of diagnostic criteria for anxiety disorders, for some autistic individuals, anxiety can also present in idiosyncratic ways, or in ways more specific to autistic populations (Kerns et al., 2014). For example, parents of autistic individuals have described anxiety being associated with challenging, self-injurious and escape behaviours (e.g. running away) (Bearss et al., 2016; Ozsivadjian et al., 2012). Furthermore, phobias may be more likely to have an unusual focus, and anxiety around changes in routine is also common (Kerns et al., 2014). Due to overlapping characteristics, anxiety-related behaviours can also be difficult to disentangle from core characteristics of autism. For example, increasing repetitive behaviours could be an indicator of anxiety (Rodgers et al., 2012). Given the possibility of idiosyncratic presentation, symptom overlap and behaviours that challenge, the identification and management of anxiety could be especially difficult for parent/carers of autistic children and young people. Indeed, qualitative interviews with parents of young autistic children in mainstream education has highlighted that the overlap between anxiety and autism characteristics can complicate parental recognition of child anxiety (Simpson et al., 2020), and emotional and behavioural problems in autistic individuals are associated with reduced parental well-being (Cadman et al., 2012; Yorke et al., 2018).

Around 50% of autistic individuals will also present with an intellectual disability (ID) (Charman et al., 2011) and the identification and management of anxiety may be especially challenging for parents of autistic individuals who speak few or no words. First, communication impairments may mean parents have more difficulty understanding the triggers for anxiety and their child’s emotions. Parents may have to rely on, sometimes subtle, contextual cues to understand their child’s behaviours and emotions (Moskowitz et al., 2013; Ozsivadjian et al., 2012). Second, common anxiety and behaviour management strategies that parents may use are likely to be less effective. Indeed, the accessibility of verbal components of traditional anxiety treatments has been questioned even for verbal autistic children, who benefit from more concrete and visual approaches (Reaven, 2011). In addition, rates of challenging behaviour are often higher in individuals with more profound ID (McClintock et al., 2003), and there is evidence that anxiety can underlie challenging behaviours in autistic individuals with ID (Rapp et al., 2005). While some research suggests anxiety may be less prevalent for autistic individuals with IQ < 70 (Salazar et al., 2015), this finding is not consistent (Simonoff et al., 2008) and could be due to a current lack of valid assessment measures for anxiety in individuals with ID (Flynn et al., 2017) or anxiety-related behaviours being misattributed to other causes (Reardon et al., 2015).

Within non-autistic populations, a large body of research has explored parental management of child anxiety. For example, parental modelling of anxiety, reduced use of collaborative parenting practices and more frequent parent involvement are associated with child anxiety (Wei & Kendall, 2014). A recent synthesis of qualitative research studies has highlighted a number of anxiety management strategies used by parents of autistic individuals. These include environmental modification, providing structure and routine, and use of behavioural management strategies such as reward systems (O’Nions et al., 2018). However, despite those with ID representing approximately 50% of the autistic population, very few of the included qualitative papers stated an explicit focus on the experiences of parents of autistic children with ID. Within the autism literature, there are high levels of exclusion of autistic individuals with ID from research (Russell et al., 2019) and it is often unclear to what extent research findings generalise to those with ID. Given that the focus of the O’Nions et al. (2018) review was parental management for both challenging behaviour and anxiety, another consideration is whether parental experience and management differs when parents attribute behaviour to anxiety compared to other causes. For example, parents may be less likely to use verbal reprimands or removal of privileges in response to behaviour they attribute to anxiety. Therefore, there is a need for further exploration of parental experience of anxiety management in populations of autistic individuals with ID.

Detailed understanding of the relationship between parental anxiety management and child anxiety can lead to the development of effective and targeted interventions which include parental components (Wei & Kendall, 2014). There is a growing literature and interest in the development of targeted behavioural parent interventions for emotional and behavioural problems in autistic children and young people (Postorino et al., 2017; Tarver et al., 2019). Yet at present, the evidence base for anxiety intervention in autism has focussed on autistic individuals with IQ > 70 (Sukhodolsky et al., 2013). Furthermore, there are very few validated outcome measures to assess anxiety that are appropriate for use for autistic individuals with ID (Flynn et al., 2017). Understanding parental experience and management strategies for anxiety in autistic individuals with ID is an important area of research to inform the development of targeted intervention and outcome measurement. This study therefore aimed to explore parental experience of anxiety identification and management in a sample of parents and carers of young autistic individuals who speak few or no words. An inductive qualitative methodology was used to ensure a data-driven approach, and in-depth understanding of lived experience. Specifically, this study aimed to address the following questions: (1) what techniques and management strategies do parents describe for anxiety-related behaviour in their child; (2) how do communication difficulties impact parental understanding and management of anxiety provoking situations and behaviours; (3) what is the impact of anxiety-related behaviours on the quality of life of autistic individuals and their families?

## Methods

This study uses data from interviews conducted with parents/carers of autistic individuals conducted in the context of a questionnaire development study. The purpose of the interviews was to understand the profile of anxiety in individuals with ID, anxiety management and the impact of anxiety on the individual and family. This current article will focus on parent discussion of the identification and management of anxiety behaviours and impact of anxiety on the autistic individual and parent and family well-being. Discussion of the profile of anxiety behaviours will be presented in detail elsewhere, in addition to detail of the psychometric validation of the new anxiety assessment tool. The study received ethical approval from the NHS Research Ethics Committee (REC): Wales REC 3 (ref: 18/WA/0139).

### Participants

Participants were recruited from an existing participant database held by the Cerebra Centre for Neurodevelopmental Disorders held at the University of Birmingham, and via online adverts and invitation from autism support groups and charities. Parent interviews were included in this analysis if they reported that their child had a clinical diagnosis of autism, was aged 4–25 years and spoke odd words only or never a word on the Wessex Questionnaire (Kushlick et al., 1973). As part of the wider study, interviews were also conducted with parents of individuals with ID without an autism diagnosis. However, to ensure the homogeneity in this sample and analysis, they are not included in this current report.

A total of 17 interviews with parents/carers of autistic children and young people (m_age_ = 14.29, SD = 5.67, range = 7–24) are included in this analysis. Participant demographics are provided in Table 1.

**Table 1.** Participant demographics.

Age (years)	Mean (SD)	14.3 (5.6)
	Min–max	7–24
Sex; n (%)	Male	15 (88.2%)
	Female	2 (11.8%)
Diagnosis; n (%)	Autism	17 (100%)
Additional diagnoses; n (%)	Fragile X syndrome Tuberous sclerosis complex	1 (5.9%) 1 (5.9%)
Parent/carer age; mean (SD)		49 (1.7)
Parent/carer sex; n (%)	Male	2 (11.8%)
	Female	15 (88.2%)
Relationship to child; n (%)	Father	2 (11.8%)
	Mother	15 (88.2%)
Household income; n (%)	Less than £15,000	1 (5.9%)
	£15,001–£25,000	22 (11.8%)
	£25,001–£35,000	4 (23.5%)
	£35,001–£45,000	2 (11.8%)
	£45,001–£55,000	1 (5.9%)
	£55,001–£65,000	3 (17.6%)
	£65,001 or more	4 (23.5%)
Highest level of parent education	No formal qualifications	0
	Fewer than five GCSEs or O-levels	0
	Five or more GCSEs or O-levels	1 (5.9%)
	Three or more A-levels	2 (11.8%)
	University degree	4 (23.5%)
	Masters or doctoral degree	6 (35.3%)
	Not provided	4 (23.5%)
SCQ score; mean (SD)		23.9 (5.6)
ADAMS; mean (SD)	General anxiety^a^	13.1 (3.8)
	Social avoidance^a^	11.2 (4.0)
	Depressed^a^	4.7 (2.5)
	Manic/hyperactive^b^	11.2 (2.3)
	Compulsive behaviour^c^	5.9 (2.5)
Wessex; n (%)	Non-verbal	4 (23.5%)
	Odd words only	13 (76.5%)

SD: standard deviation; SCQ: Social Communication Questionnaire; ADAMS: Anxiety, Depression and Mood Scale. N.B.: specific data on participant ethnicity were not obtained.

a Maximum subscale score = 21.

b Maximum subscale score = 15.

c Maximum subscale score = 9.

### Procedure

Parents responding to study adverts were contacted by a member of the research team to assess eligibility and provide further information about the research. Eligible families were sent a link to complete online consent forms and questionnaires, and a time was arranged to complete the interview. Interviews were conducted over the phone, by a member of the research team (J.T., G.E. or A.S.). Telephone interviews are increasingly being used as a method of qualitative data collection and are an effective form of interview format when face-to-face interviews are not feasible (Gubrium & Holstein, 2001). All interviewers were trained in interview administration prior to data collection to ensure consistent interview style. Interviewers did not have any previous relationship with interview participants, but participants were aware of the aims of the research project and the interviewer’s involvement in the study. Participants were advised that their interviews were being recorded, but comments would remain confidential, and identifiable information would be removed from interview transcriptions. Interview length varied from 28 to 120 min, with a mean length of 55 min.

The main aims of the interview were to understand more about the profile of anxiety in individuals with autism and/or ID who speak few or no words. The interview schedule was developed as part of a previous unpublished research project to profile anxiety in individuals with ID. The interview consisted of 20 questions and open-ended prompts; examples are provided in the Supplementary material.

Interviews were transcribed verbatim by members of the research team (J.T., P.M., L.P.). For interviews transcribed by other members of the research team, the author leading on analysis (J.T.) spent more time in phase 1 of data analysis (familiarisation) and also checked transcriptions back against the original recordings for accuracy.

### Measures

To characterise the sample, parents/carers completed the Social Communication Questionnaire (SCQ) (Rutter et al., 2003), the Wessex Questionnaire (Kushlick et al., 1973) and the Anxiety, Depression and Mood Scale (ADAMS; Esbensen et al., 2003) as measures of autism characteristics, degree of ability and anxiety/mood. Further details about these measures are provided in the Supplementary material.

### Data analysis

In order to address the research questions, throughout the analysis, any data related to parental recognition and management of anxiety-related behaviour, and the impact of anxiety on the individual and wider family were coded. The aim of the analysis was to provide a detailed and nuanced account of themes identified in the data. While the aim of this research project was to address specific research questions, no existing coding framework was utilised for coding analysis. Instead, the focus of the analysis was a largely inductive approach to identify themes closely related to the data (Thomas, 2006).

Analysis followed the step-by-step approach to thematic analysis proposed by Braun and Clarke (2006). This approach was selected due to its flexibility, allowing inductive and deductive analysis to assess manifest and latent content (Joffe, 2011). First, the lead author (J.T.) established familiarity with the data and line by line semantic coding was conducted (Boyatzis, 1998). An initial coding manual was created containing code names, and brief descriptions and examples of each code. During this initial coding, codes were placed under a-priori grand themes (identification of anxiety, anxiety management and impact of anxiety); these themes were chosen based on the aims of this research project and questions in the interview schedule. Following this, each interview was independently coded by two authors (J.T. and E.P.), using the coding manual. During coding, surrounding data were kept around each code of interest to ensure context was not lost from the participant narrative (Bryman, 2001). Following completion of the independent coding, the authors discussed coding, discrepancies in coding were agreed by consensus and refinement to the coding conducted. Codes were then reviewed and placed into meaningful groups and themes by the lead author (J.T.). Next, a thematic map was developed to identify the main overarching theme and key themes that fit into each overarching theme. The themes and included codes were reviewed in multiple team discussions to ensure interpretation was not restricted to the views of a single author. At this point, themes were refined and further defined, a narrative was drafted, and key quotes were identified that most appropriately described each theme.

### Community involvement

A parent advisory group of six parents representing children and adults with ID have provided feedback on the design and methodology of the wider questionnaire development study from which these qualitative data are drawn.

## Results

A total of 15 themes were identified in the analysis which were placed under three a-priori grand themes to address study aims. Three themes (communication; overlap with other behaviour; knowing the person well) were placed under the grand theme of parental recognition of anxiety. For parental management of anxiety, six themes described management strategies (the balance between avoidance and exposure; sensory; preparation for upcoming events; distraction; time and space; reassurance). Parents also described a number of factors related to the effectiveness of strategies: timing; background factors; and availability of strategies. Finally, three themes were identified under the grand theme of anxiety impact (restricted world; parental well-being; impact on health).

### Parental recognition of anxiety

#### Communication

As expected, parents often discussed difficulty understanding the anxiety due to their child’s limited communication, including the fact their child uses few or no words, and may also show more limited use of non-verbal communication such as facial expressions. Because of this, parents discussed being reliant on behavioural manifestations and making interpretations of their child’s emotions based on behaviour. Parents expressed that reliance on behavioural manifestations can be difficult, and discussed concern that they may misinterpret behaviours:

Because he is sort of non-verbal and not able to express, everything is always in (name)’s head, so unless he is showing something behaviourally, or is showing outward signs, you just don’t know, you just don’t know what is going on in the little boy’s mind. (Mother of 12-year-old male)

#### Overlap with other behaviour

When relying on behavioural manifestations, parents also described the difficulty in teasing apart behaviour associated with anxiety from other behaviours or forms of distress. Most commonly, parents described anxiety behaviours that overlap with characteristics of autism (e.g. rocking, sensory seeking, repetitive speech) which consequently made it difficult to understand whether the behaviour is the result of anxiety, or an autism characteristic. Parents also discussed overlap with other behaviours including hyperactivity and those associated with pain and illness:

He’s got the autism as well and sometimes it’s very difficult to understand whether it’s an autistic behaviour, or whether it’s behaviour caused by anxiety, or both. (Mother of 19-year-old male)

Parents may be dependent on demeanour, or the speed, frequency or intensity of behaviours to help them understand whether they are the result of anxiety. Some parents also discussed using and collating situational information to help them understand whether behaviours are anxiety, and what the trigger is:

you know you just get clues like that and you can try and piece them together a bit to try and understand what might be causing something . . . you have to try and watch, and observe and piece together really. (Mother of 7-year-old male)

#### Knowing the individual well

Linking this together, and what became apparent in some parents’ narratives, is a sense of needing to know the individual very well in order to recognise and respond to anxiety. Some parents reflected back on when their child was younger, and not recognising anxious behaviours or triggers of anxiety the way they do now they better understand their child:

I would say they (anxiety behaviours) came on fairly gradually, it’s taken us a number of years I suppose to understand that’s what they are. (Mother of 23-year-old female)

### Parental management of anxiety

Parents described a number of strategies that they use to help manage the anxiety and its impact. The strategies that parents used largely fell into two main areas: preventive/long-term strategies; and more reactive or short-term strategies being used in response to anxious behaviour in the moment.

#### Long-term/preventive strategies

##### The balance between avoidance and exposure

Many parents in the interviews discussed avoiding situations, circumstances or things likely to trigger anxiety in order to prevent or reduce its occurrence. In order to protect their child, parents discussed not wanting to place them in situations likely to cause them extreme or unnecessary distress, and where possible, would consequently avoid it. Understandably, avoidance of triggers was especially discussed in relation to situations likely to be very distressing, or those likely to result in challenging behaviour such as aggression or meltdowns; indeed all 17 parents stated that anxiety is associated with some form of challenging behaviour. Avoidance was also often discussed in situations that their child finds very aversive due to sensory processing difficulties (e.g. hand dryers):

We don’t do that, sort of put him in a situation, because you just have to know that’s not going to be safe, so you know, you withdraw, you just don’t escalate these things. (Mother of 18-year-old male)

However, in some interviews, there was recognition from parents that avoidance of situations may not always be the most optimal strategy to manage anxiety in the long-term. For example, parents discussed how some situations that used to trigger anxiety no longer do, since repeated exposure to anxiety triggers has provided opportunity to ‘get used to it’. Some parents discussed strategies such as purposefully exposing their child to situations that cause anxiety (e.g. using various routes to destinations) to prevent anxiety later if the trigger cannot be avoided:

We’ve learnt our lesson now, when you go somewhere new for the first time, the second time you go, you try it on a different route, or you do something different. Because you’re almost setting yourself up to have problems in the future if you try to keep things the same. That’s one of the things I’ve learnt across the years. (Mother of 11-year-old male)

Parents also discussed the need to try and expose their child to anxiety provoking situations if possible, to ensure their world is not restricted and that they can engage in activities they would enjoy if they could get past the initial feelings of anxiety. However, there was acknowledgement from one parent that this is not an easy thing for parents to do, requiring determination and willingness. Indeed, the management of anxiety-related behaviours is likely to be very complex, and there are multiple factors besides willingness and determination that mean parents may not feel able to do this:

It took him about 4 times, and now it is the best thing you can give him . . . but you have to go through this (behaviour); if you haven’t got determination and willingness to take the hits . . . he would never experience anything. (Mother of 24-year-old male)

#### Sensory strategies

The majority of parents also discussed the importance of understanding sensory needs and sensory strategies, in order to manage anxiety. This included a number of different strategies such as deep pressure and massage, the use of ear defenders or reducing noise in the environment, and the use of a backpack with light weights. Parents described how use of these strategies, which parents encouraged using, or which autistic individuals requested or sought out, help to regulate their child, thereby reducing anxiety and anxiety behaviours:

I think this is termed like a sensory diet, if he doesn’t have enough opportunity to express and release that, I think he gets very anxious. (Mother of 7-year-old male)

#### Preparation for upcoming events

Another commonly used strategy that parents discussed was preparation for upcoming events or transitions. Most commonly, this included the use of visual schedules or pictures, or verbal repetition of upcoming activities during the day. Overall, parents were less clear whether this strategy actually led to a reduction in anxiety. However, they continued to use it to aid their child’s understanding of upcoming events:

We’ve started recently in the past year trying to tell him what’s going to be happening before going doing something and also showing him little text cards and that’s helped a little bit. (Mother of 11-year-old male)

In addition to preparing their child for upcoming events that may cause anxiety, some parents also discussed the need to prepare themselves, or plan ahead. This included conducting detailed ‘risk assessments’ that armed parents with plans of where they can go, and what they would do if anxiety arises that would help them to manage in the moment:

I am risk assessing where it is appropriate to go . . . and what is safe. I am a single parent, so I have to really think, where we can go, where we can manage and what’s safe and as part of that risk assessment I think will there be any dogs. So every time I go out, I consider the difficulties that may arise to minimise the anxiety. (Mother of 19-year-old male)

#### Dealing with anxiety in the moment

When managing anxiety ‘in the moment’, parents described using a number of strategies, mostly aimed at calming the situation and reducing the impact of anxiety.

#### Distraction

The strategy most commonly discussed to manage anxiety in the short-term was distraction. Distraction included a number of techniques such as talking about their child’s interests or giving a favourite or preferred item, playing games or going for a walk in the park:

If I can see it coming, and I know what the cause of it is, and there’s a way around it I can maybe put some kind of distraction in, then I can calm him down enough for it to be less of an impact. (Mother of 12-year-old male)

#### Time and space

When their child was feeling anxious, parents described that the best way to calm the situation was to back away from their child, allowing time and space to calm themselves down. Parents often described anxiety manifesting in the form of high risk or challenging behaviours, and, provided the safety of their child was ensured (e.g. move away from furniture or large objects) providing time and space is the best way to deescalate a situation that has caused anxiety. Some parents also discussed how important it is to not use many words during this time, which autistic children and young people may find hard to process:

If you overwhelm him with verbal information, you know, like with a (non-autistic) child you might kind of say, you might offer lots of information through speaking to them and say ‘Oh come on don’t worry’ . . . I think doing that just kind of overwhelms him even more. (Mother of 11-year-old male)

#### Reassurance

Naturally, parents also often described using reassurance in order to comfort their child when they are feeling anxious. Sometimes, parents did not provide much explanation of what they meant by reassurance. However, where parents did expand on this, reassuring their child when they felt anxious was done using a variety of methods including cuddles and physical comfort, verbal reassurance including encouragement, explanation and reassuring phrases such as ‘it’s ok’; one parent also described the use of soothing noises.

#### Effectiveness of strategies: the complexity of anxiety management

Despite all parents describing the use of a wide range of anxiety management strategies, parents also described a number of factors that can impact their effectiveness. These factors, described below, highlight the complexities that parents can face when helping their child to manage anxiety.

#### Timing

The timing at which the strategy is implemented is one such factor; parents described the need to make sure that ‘in the moment’ strategies are implemented early enough to prevent an escalation in anxiety that would be harder to manage/reduce. Timing was also discussed particularly in relation to preparation, including the timing at which schedules are presented before the transition. Parents need to provide enough time to process, but not too long that the individual may forget, or become more anxious due to possible rumination. One parent also described that preparation for an event is less effective if it is delivered at a time when the child is already feeling anxious, stating that their child is almost too ‘wobbly’ to take in the information:

I think as long as (name) is prepared and again there is a balance in that, so the timing’s important, so if we do it too early then that provokes some anxiety, but obviously he needs to be adequately prepared, so usually we start talking about it three or four days before something is going to happen. (Mother of 21-year-old male)

#### Background factors

Other times, parents also alluded to the impact of other background factors which mean no matter what they do, the anxiety will just come out, or their usual strategies are much less effective. Some parents stated awareness of other additional background factors such as hunger, tiredness or hormones that they felt played a role in the effectiveness of anxiety management. Other times, parents did not state a specific background factor, instead suggesting that there are periods of time, or days where anxiety is more severe or more difficult to manage, perhaps due to factors unrecognised by the parent:

But I have to admit, every now and again, sometimes, it’s almost like it doesn’t matter what we do it’s going to happen, the meltdown thing is rising and we’re just, we’re almost delaying the inevitable. (Mother of 11-year-old male)

#### Availability of strategies

Due to the nature of some of the strategies, at times, it is just not possible for parents to implement them. For example, access to distraction techniques may not be possible if tangible items are not available. Other strategies, such as avoidance, may also not be possible. Parents recognised that some aspects of life are outside of their control; it is not possible to completely manage exposure to triggers:

At the minute the only strategies he’s got are, having a bath, getting his PJs on and going on the IPad, but obviously when we’re out, those aren’t realistic options, so at the minute he can’t really control his anxiety. (Mother of 7-year-old male)

### Impact

Despite the array of management strategies that parents described, the impact of anxiety on the quality of life of the autistic individual and their families was evident in all interviews.

#### Restricted world

For both parents and autistic individuals, anxiety prevents engagement in activities that they would like to do both individually and as a family. For autistic individuals, parents described how anxiety can prevent engagement with others, community activities and hobbies. Where parents were able to support their child to engage in activities, there was a sense that engagement remains limited and difficult for parents. For example, a lot of forward planning is needed (as described in theme above), there are very few places that the family can go, and not all members of the family can go together. Interestingly, parents often described times that should be especially enjoyable (e.g. family holidays, school holidays and Christmas), as being particularly difficult times due to anxiety associated with changes in routine. Siblings missing out on opportunities and experiences, such as not being able to have friends over, was also discussed by some parents:

It’s restricting what we can do as a family, because we can’t really do anything, unless it’s going for a walk in the park or a walk in the countryside, we can’t really do anything as a family of 4, because (name) just can’t cope with going to places. We can’t go round to friends’ houses, it’s very, very restricting. (Mother of 7-year-old male)

#### Parent well-being

Parents also discussed the impact of child anxiety on their own well-being. Parents discussed feelings of worry, stress and anxiety about their child’s distress, and wanting to make sure their child is happy. Parents also discussed worry and concern about their ability to detect or manage the behaviours associated with anxiety. Some parents particularly discussed this concern as their child gets bigger and stronger, and they may be less able to physically manage behaviour associated with anxiety. Two parents also acknowledged the need to manage their own stress and anxiety around their child, in case they ‘pick up’ on parental distress, and it works to exacerbate child anxiety:

I just, worry for them all the time, and all you want to do is for your child to be happy. . . And when your child displays the anxiety behaviours that I have seen, there’s clearly something that is making him anxious and I can’t help him with, so for me, there’s a constant worry that I’m not being the super parent that I aspire to be. (Mother of 19-year-old male)

#### Impact on health

Finally, some of the parents discussed concerns about the impact of anxiety on their child’s physical health. Often, this was apparent because of the very physical reactions to anxiety that their child has shown, for example, stress seizures or extreme weight loss. Some parents also discussed concerns about how anxiety and anxiety-related meltdowns must be taxing and exhausting for their child. One parent discussed how their child’s access to health services has been impacted; physical health services had refused care because of the anxiety-related behaviours that were shown in healthcare settings.

## Discussion

This study aimed to explore anxiety recognition and management in parents of autistic individuals who speak few or no words. The study revealed 15 themes which fit under three a-priori, overarching themes related to parental recognition of anxiety, management of anxiety and anxiety impact.

Interviews identified parents face particular challenges recognising anxiety. Parents discussed how reduced use of communication and overlap of anxiety behaviours with autism characteristics and other markers of distress can complicate their assessment of their child’s behaviour and emotions. This finding is consistent with another recently published qualitative exploration of parental perceptions of anxiety in a sample of parents of young autistic children in mainstream education (Simpson et al., 2020), where parents discussed overlap between anxiety and autism characteristics complicating their assessment of anxiety. Indeed, even in verbal autistic children, the presence of alexithymia may complicate assessment of anxiety and internal states (Shah et al., 2016). This finding therefore seems to be consistent across the autistic spectrum, although future research would need to confirm this by conducting interviews with parents of individuals both with and without ID. Parents reported needing to know their child very well, and conduct in-depth assessment of contextual cues to understand possible functions of behaviour. This highlights potential challenges and has implications for autistic individuals in care or hospital settings. High staff turnover, for example, may prevent staff developing detailed understanding of functions of behaviour, and changes in baseline behaviour which could indicate underlying mental health issues (Hatton et al., 2001).

In terms of management strategies, parents described using a number of strategies to prevent and manage anxiety should it occur. Interestingly, a number of strategies parents reported using, such as preparation and sensory calming strategies, overlap with components of behavioural parent-led interventions for autistic children (Bearss et al., 2015; Hallett et al., 2020). Graded exposure, which some parents reported use of, is also included in such interventions, and is a key component of anxiety interventions for autistic and non-autistic children with an established evidence base (Öst & Ollendick, 2017; Sukhodolsky et al., 2013). However, sensory processing difficulties mean autistic individuals may find some situations particularly aversive. Furthermore, high rates of challenging behaviour have been associated with anxiety both in the current study and elsewhere in the literature (Rapp et al., 2005). Exposure is therefore a component of intervention that would require careful management in autistic individuals with ID; parents and carers may need additional support to help manage exposure-based tasks.

Some parents also described avoiding situations their child is likely to find anxiety provoking. While a likely effective short-term strategy, avoidance may not be the most optimal strategy for long-term anxiety management. Avoidance can work to inadvertently maintain the anxiety cycle, by removing opportunities for the individual to obtain mastery experience in overcoming anxiety. Consequently, anxiety interventions usually aim to reduce anxiety-related avoidance (Piccirillo et al., 2016). In a similar vein, reassurance, another strategy that parents discussed, is also a strategy that has been implicated in some models of anxiety maintenance (Pincus et al., 2008). As was evident in the interviews, avoidance of situations can lead to reduced opportunity to engage in community and recreational activities, impacting quality of life. In order to improve outcomes for autistic individuals and their families, societal changes such as expanding the availability of autism-friendly events, may help parents and autistic individuals feel more able to engage in community and recreational activities (Billstedt et al., 2011).

Despite parents reporting the use of management strategies that overlap with components of evidence-based treatments, all parents described anxiety as something that continues to be problematic, impacting the quality of life of the autistic individual and that of their wider family. This is in keeping with other research reporting a bidirectional relationship between child emotional and behavioural problems and parent/carer well-being in autism (Yorke et al., 2018). Early, targeted interventions are likely needed for parents/carers of young autistic children, as well as psychoeducation for both parents and healthcare professionals to identify behaviours associated with anxiety. Anxiety should be routinely assessed during clinical assessments, including assessment of change in behaviour from a person’s baseline levels. The development of valid assessments for anxiety for autistic individuals with ID is also an important area for future research (Flynn et al., 2017).

This study has a number of limitations important to consider during the interpretation of findings. First, conducting an IQ assessment to confirm the presence of ID was beyond the scope of this study. We used parent-report of verbal language ability as a proxy measure of ID based on research showing high levels of overlap between verbal language use and ID in autism (Bal et al., 2016). However, it is important to note that non-verbal autistic individuals can also present with IQ in the normal range (Bal et al., 2016). Despite this, the focus of this study on minimally verbal autistic individuals is a notable strength, given selection bias against those with ID or less frequent language use in autism research (Russell et al., 2019). Another possible limitation is that parents/carers in this study were highly educated; the majority of parents reported having a postgraduate level university degree. Findings may therefore not represent the views of parents/carers of all minimally verbal autistic individuals, as is often the case in qualitative research with relatively small sample sizes. Future research should consider exploring the views of parents in a more representative sample of parents/carers of autistic individuals who speak few or no words. Finally, while this study focussed on parent/carer views of anxiety, future research should also look to obtain the viewpoints of autistic individuals. Communication resources, such as Talking Mats, are an effective way to allow individuals with ID, or those who speak few or no words, to express their views and could be utilised in future research designs (Murphy & Cameron, 2008).

## Supplemental Material

Description_of_measures_used – Supplemental material for Anxiety in autistic individuals who speak few or no words: A qualitative study of parental experience and anxiety management Click here for additional data file. Supplemental material, Description_of_measures_used for Anxiety in autistic individuals who speak few or no words: A qualitative study of parental experience and anxiety management by Joanne Tarver, Effie Pearson, Georgina Edwards, Aryana Shirazi, Liana Potter, Priya Malhi and Jane Waite in Autism
