# Supplementary material for: Anxiety in autistic individuals who speak few or no words: A qualitative study of parental experience and anxiety management
Source: Autism. 2020 Oct 1;25(2):429–39. doi: 10.1177/1362361320962366 (PMC7874371; doi:10.1177/1362361320962366)
Supplement: Description_of_measures_used – Supplemental material for Anxiety in autistic individuals who speak few or no words: A qualitative study of parental experience and anxiety management [file Description_of_measures_used.pdf]

**Description of measures used**

*Social communication questionnaire (SCQ; Rutter et al., 2003).*

The SCQ current version is a parent-report measure and a screening tool for autism characteristics. It consists of 40 items presented in a yes/no format and form 3 subscales; communication, social interaction and repetitive and stereotyped behaviours. A cut-off score of >15 indicates possible ASD and a score >22 indicates possible autism. The SCQ has high levels of specificity (.60-.67) and sensitivity (.75-.96), good concurrent validity with the ADI and Autism Diagnostic Observation Schedule (ADOS) and good internal consistency (Berument et al., 1999).

*The Wessex Behaviour Scale (Kushlick, Blunden, & Cox, 1973)*

The Wessex Behaviour Scale consists of 15 items presented on a Likert scale, that measures the degree of ability in individuals with intellectual disability. It assesses mobility, self-help skills, continence, reading, writing, verbal ability and vision which are summed up into a 'Social and Physical incapacity score and a 'Speech, self help and literacy' score. The measure has good inter-rater reliability at .62 (Palmer & Jenkins, 1982).

*Anxiety, Depression and Mood Scale (ADAMS; Esbensen et al., 2003)*

The ADAMS is an informant questionnaire that includes 28 items on four-point rating scales from 'not a problem' to 'severe problem'. There are five subscales of manic/hyperactive behaviour, depressed mood, social avoidance, general anxiety and compulsive behaviour which have maximum subscale scores of 15, 21, 21, 21 and 9 respectively. The scale has been validated for individuals with intellectual disabilities aged 10-79 and has good test-retest reliability for the scale at .81.

| Supplementary Table: Example questions from interview schedule |
|---|
| • I am interested in any difficult or negative emotions that (X) may experience. Can you tell me about them?<br>• Does (X) ever get anxious, worried or apprehensive?<br>• Have you noticed any patterns (when anxiety occurs)?<br>• Is there anything that you can do that helps to reduce (X)'s anxiety?<br>• How does the anxiety affect (X) day to day?<br>• How does (X)'s anxiety affect you and your family? |
